# Physical Activity Barriers, Facilitators, and Preferences in Rural Adults with Obesity

**DOI:** 10.1007/s12170-024-00754-5

**Published:** 2024-12-04

**Authors:** Nashira I. Brown, Peter Abdelmessih, Laura Q. Rogers, Dori Pekmezi

**Affiliations:** 1 Department of Health Behavior, University of Alabama at Birmingham, Birmingham, AL, USA; 2 Department of Medicine, Heersink School of Medicine, University of Alabama at Birmingham, Birmingham, AL, USA

**Keywords:** Environment, Psychosocial, Health Behavior, Weight

## Abstract

**Purpose of Review:**

To identify physical activity-related barriers, facilitators, and preferences among adults with obesity living in rural areas, a scoping review was conducted.

**Recent Findings:**

Ten studies conducted in the United States, Australia, Nepal, and Mexico were included. The barriers reported most often were social/physical environment concerns (e.g., lack of resources/social support), as well as finding time. The one study comparing barriers across obesity classes I-III found minimal differences. Th most frequently reported facilitators included fitness trackers and social interaction/being part of a group. In the only study reporting intervention preferences, residents of rural areas preferred web-based delivery, whereas individuals with obesity favored face-to-face delivery.

**Summary:**

When developing physical activity interventions, it is important to consider environmental and psychosocial factors that can hinder or facilitate engagement among rural adults with obesity. More research on preferences is needed to inform future interventions.

## Introduction

Obesity and physical inactivity are more prevalent in rural United States (U.S.) regions compared to urban and metropolitan areas [1, 2]. Correspondingly, these areas of open countryside, with sparse population densities of less than 2,500 people, have higher death rates from related chronic health conditions such as cancer, heart disease, and chronic respiratory disease, with increasing rural-urban differences over recent years [3, 4]. Environmental factors (e.g., limited places to be active), economic factors (e.g., low income levels), and social factors (e.g., lack of support from family and friends) [5] may contribute to this health disparity [6].

To address this disparity, several physical activity interventions have been tested (e.g., in-person, web-based, telephone-based) in adults with overweight and obesity residing in rural regions across the US, mainly in the southern regions. A recent systematic review of physical activity interventions [7] reported promising findings including significant increases in recreational physical activity and exercise [8, 9] and improvement in determinants of behavior change (e.g., increased self-efficacy, moving from contemplation to action/maintenance stage) among rural adults with overweight and obesity [10, 11]. Despite these favorable findings, no studies reported on barriers, facilitators, or preferences related to the interventions which could have impacted engagement and success among participants. Evidence shows that this at-risk population does improve their levels of physical activity in response to interventions. However, scaling up to address the public health concern might require a better understanding of their barriers, facilitators, and preferences related to physical activity [12–15].

Previous research suggests that individuals with obesity face additional physical activity challenges [16] and experience less benefit from conventional physical activity interventions in comparison to adults without obesity [17]. One study conducted among non-rural Latina women with overweight (42%) and obesity (38%) found that after a 6-month physical activity intervention, participants with overweight reported engaging in an average of nearly 30 additional minutes per week compared to those with obesity [17]. While this research suggests that physical activity interventions may be less effective for adults with overweight and obesity who do not reside in rural areas, it is likely that rural adults with obesity face greater challenges in being active. The interplay of barriers to physical activity in rural areas, along with obesity-related health issues such as pain and physical discomfort, warrants further examination. In addition to barriers, it is also vital to understand the facilitators and preferences. These findings have important implications for researchers and healthcare professionals, particularly in areas with higher body mass index (BMI) and inactive rural regions, highlighting the need for further investigation.

To our knowledge, recent research has focused on either obesity or rural health, without exploring physical activity barriers, facilitators, and/or preferences in rural adults with obesity [18, 19]. Hence, this scoping review aims to synthesize the current literature on physical activity in this vulnerable population and pinpoint areas needing further exploration. These findings will provide a valuable insight for developing targeted physical activity interventions and will suggest directions for future research in this field.

## Methods

### Search Strategy

This review adhered to the PRISMA Extension for Scoping Reviews (PRISM-ScR) [20]. A systematic search was performed in the following databases: PubMed, Google Scholar, Web of Science, and Embase. The databases were first searched on August 15, 2021, with an updated search on March 1, 2024. The following terms were used for the search: physical activity (sport, exercise, recreation), adult, rural (rural population), barriers, facilitators, preferences (attitude, perception, opinion, beliefs), obesity (obese).

### Eligibility Criteria

Studies were included if they met the following criteria: (1) participants were adults (≥ 18 years of age), 2) participants resided in rural area; or study included a rural sample for comparison, (3) the sample consisted of individuals with obesity (BMI ≥ 30 kg/m^2^) or included at least 50% of participants with a BMI ≥ 30 kg/m^2^ if the study did not solely involve participants with obesity; or if less than 50% of the sample did not have a BMI ≥ 30 kg/m^2^, outcomes by BMI category were reported, (4) barriers, facilitators, and/or preferences related to physical activity were reported, (5) the study was a quantitative, qualitative or mixed methods, and (6) published 2011 through 2024 to reflect the latest trends in this literature. The following were excluded: case reports, conference and meeting abstracts, protocol papers, reviews and articles not published in English.

### Study Selection

Articles from the search were imported to EndNote, where duplicate entries were removed, and the final list was exported to Microsoft Excel. Titles and abstracts were reviewed independently reviewed for potential eligibility (NIB, PA). For studies not screened out by abstract/title review, two investigators (NIB, PA) reviewed the full-text articles against inclusion and exclusion criteria and made final determinations. There were no disagreements. Citation searching, both backward and forward, was performed using SpiderCite, an automated tool [21].

### Study Data Extraction

The following data were extracted and presented for each included article: lead author, year of publication, country, methods, participant and study characteristics (i.e., sample size, gender, age, BMI, prevalence of obesity in study sample, and reported comorbidities), rural characterization, BMI comparisons, rural comparisons (i.e., to urban participants, if sample was not purely rural), intervention characteristics (if there was an intervention), and physical activity barriers, facilitators, and preferences. For ease of reporting and interpretation, the findings for barriers, facilitators and preferences were classified into four overarching categories: ecological, psychological, physical and demographic [18].

### Data Analysis and Reporting

The combined study characteristics, participant characteristics, and barriers, facilitators and preferences data of the included studies were descriptively analyzed using frequencies and means. These results are presented in a descriptive format.

## Results

### Study Selection

**Fig. 1:**
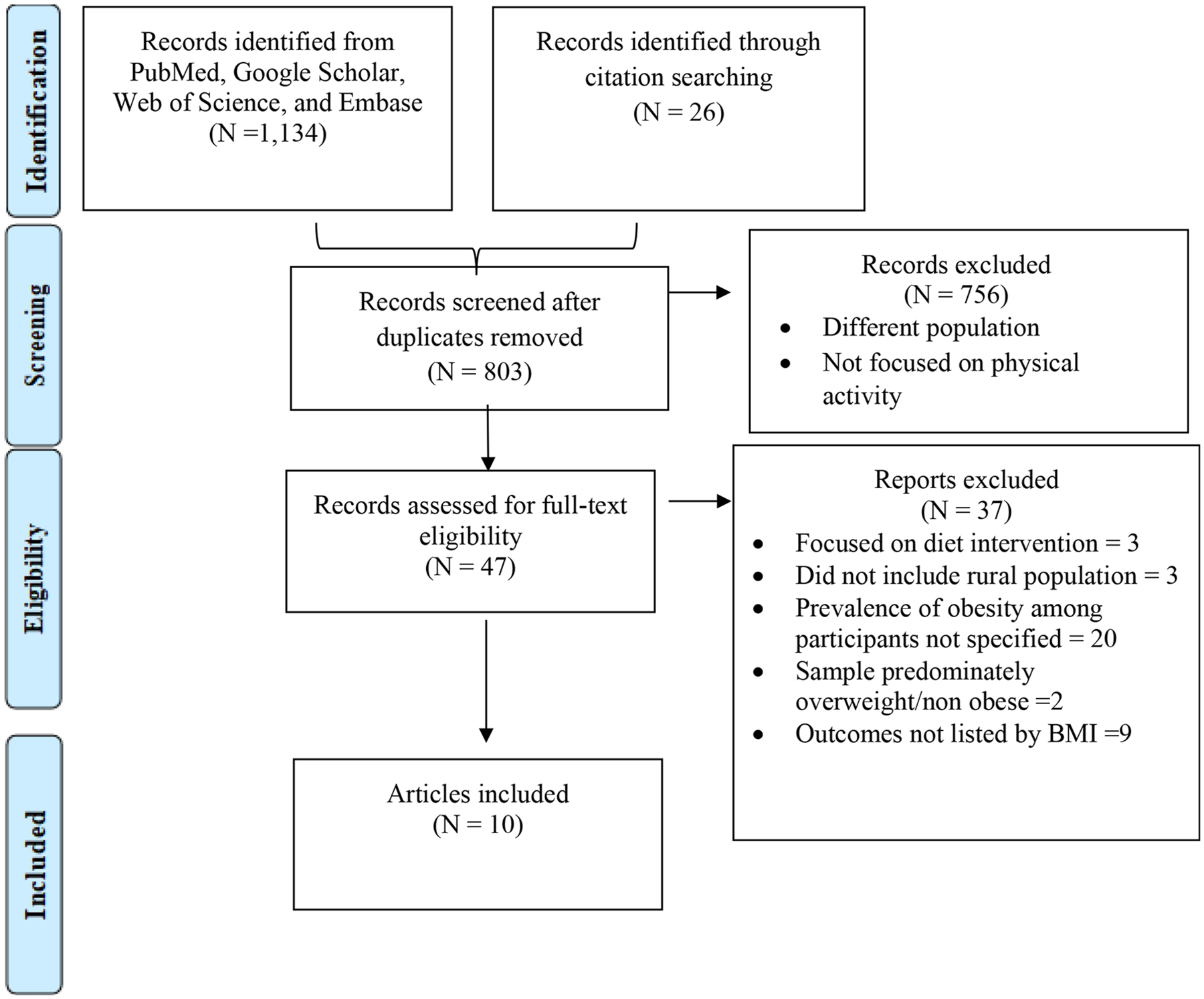
PRISMA Flow Diagram

A total of 1,134 citations were retrieved, then 803 titles and abstracts were assessed before reviewing 47 full texts. Following additional exclusions (e.g., sample was predominately non-obese or overweight), 10 articles were included as shown in Fig. 1.

### Study Characteristics

Table 1 provides a summary of the study characteristics. Most of the studies were conducted in the United States [22–27], with two conducted in Australia [28, 29], one in Nepal [30], and one in Mexico [31]. Of the 10 studies, eight were cross-sectional [22–25, 28–31], and two were pilot studies (single arm pre/post-test design) [26, 27]. Mixed methods designs were used in the two pilot studies [26, 27] and one cross-sectional study [23]. The cross-sectional studies included interviewer administered and self-administered surveys, telephone interviews and anthropometrics collected concurrently [22–25, 28–31]. The two pilot studies also included self-administered surveys and involved interventions lasting three weeks each [26, 27]. The mixed methods design studies involved semi-structured interviews [26, 27] and self-administered surveys with open and closed-ended questions [23].

### Participant Characteristics

Participant characteristics are presented in Table 1. The aggregate sample size from the 10 studies was 11,687, with a median of 441 (range: 8 − 6,406). Most studies (*N* = 9) included adults with a mean age greater than 50 years. The gender distribution across the studies was 62.8% female and 37.2% male. Two studies included only females [23, 29], while one study included males only [26].

Eight of 10 studies provided the average BMI for their samples, which was 31.3 kg/m^2^ (range: 24.26–35.9) [22–24, 26–30]. Most studies (*N* = 6) provided overall obesity prevalence [23, 25–31]. In each sample, an average, 50% of participants had obesity. Six studies reported participants’ comorbidities, and diabetes (i.e., type II and previous gestational) and hypertension were most frequently mentioned conditions [25–27, 29–31]. Osteoarthritis [27], dyslipidemia [27], polycystic ovary syndrome [29], and sleep apnea [27] were other reported comorbidities. A single study reported 46% of participants had a chronic illness though exact conditions were not specified [28].

### Barriers

Seven studies reported on perceived barriers for physical activity [22–24, 26, 27, 29, 31]. The majority of reported barriers were ecological barriers and consisted of environmental (*N* = 4, e.g., lack of places to be active), and weather (*N* = 1) challenges [22, 24, 26, 27, 29]. Following ecological barriers, were psychological barriers including difficulty finding time (*N* = 2), depression (*N* = 1), lack of self-discipline (*N* = 1), lack of motivation (*N* = 1), and lack of social support [23, 27, 29, 31]. Physical health barriers were the least assessed with two studies reporting knee issues (*N* = 1) and fatigue (*N* = 1) [23, 26]. Three studies examined the relationships between BMI and barriers [22, 24, 29]. Adachi and colleagues found that environmental concerns, particularly difficulty walking and unattended dogs in the neighborhood were related to increased BMI [22]. Likewise, the other two studies found increased BMI was linked to negative perceptions of community environmental factors (e.g., a lack of available spaces to be active, few sidewalks and bike lanes, and limited affordable facilities) [24, 29].

One study explored distinctions in physical activity barriers in women across obesity classes I-III) employing mixed methods which included open and close-ended survey items [23]. The close-ended questions with limited, pre-defined response options revealed a consistent report of inadequate self-discipline in each obesity class. In contrast, the open-ended approach identified themes related to lack of time in addition to physical factors (e.g., weight, knee issues), psychological factors (e.g., lack of motivation), social factors (e.g., lack of social support/being alone), and weather-related factors (e.g., too hot or cold outside) [23]. Across obesity classes I-III, no overall differences in barriers were found.

### Facilitators

Few studies (*N* = 4) reported on physical activity facilitators [24, 26, 27, 30]. The most frequently cited physical activity facilitators were psychosocial and ecological factors (i.e., socio-ecological) which included activity monitors (*N* = 2) and social interaction/being part of a group (*N* = 2) [26, 27]. Two studies conducted among older rural men found that social interaction and group environment were motivating aspects important for physical activity interventions [26, 27]. Additionally, one of these studies among older men noted that weather, particularly, “better weather” would allow for consistent exercise [27]. Psychosocial and physical barriers were least reported with only reports from two studies [25, 30]. Regarding demographic facilitators, one study conducted in adult patients with type II diabetes examined the determinants of adherence to PA engagement and reported patients who had a type II diabetes family history and greater socioeconomic class as more adherent to physical activity after receiving advice from a physician or others [30]. As for physical facilitators, a single study found a difference between motivation for weight loss opposed to exercise [25]. Participants motivated to lose weight were twice as likely to be individuals with obesity, African American, and female [25].

### Preferences

With only one study reporting on preferences, this was the least explored aspect of physical activity. In that study, in adults with overweight (36%) and obesity (30%), those with obesity had a preference for interventions delivered face-to-face instead of group, print, and web-based interventions [28]. However, analyses by location suggested web-based preferences were positively linked to living in a rural area. In contrast, a preference for group-based programs (involving interaction with others in a group setting, led by an instructor) was negatively linked to both obesity and having a separated marital status, but positively linked to living in regional area or town. Therefore, those living in rural areas who were not separated (e.g., married or partnered) preferred web-based interventions, whereas face-to-face interaction was preferred by individuals with obesity.

## Discussion

When taking part in standard physical activity interventions, adults with obesity may face specific challenges due to excess body weight and may not be as successful as individuals with lower BMI [17, 32]. Furthermore, these difficulties may be exacerbated for those living in rural areas as a result of environmental barriers (e.g., limited space/facilities for physical activity), potentially contributing to the worsening of rural health disparities. No reviews have identified physical activity barriers, facilitators and preferences among rural adults with obesity. Thus, we conducted a scoping review of the current research on the physical activity-related needs of this population (e.g., social support, integration of activity monitors) and identified future directions for intervention research (e.g., applying a mixed methods strategy to better understand physical activity preferences).

Overall, barriers were the most investigated and reported factor for physical activity. Our findings show the primary physical activity barriers were environmental factors and a lack of time. There are consistencies and differences with a previous review and study focused on adults with obesity or rural dwelling adults [18, 19]. Our findings are similar to the findings from a recent systematic conducted by Baillot et al. among adults with obesity, which found pain or physical discomfort, lack of time, and lack of self-discipline/motivation, as the three most reported barriers to physical activity [18]. Seguin and colleagues reported social norms, limited time, and distance from or lack of facilities as top barriers among mostly rural adults with overweight or obesity, who considered themselves as active or very active [19]. When identifying differences in barriers by obesity class, we found that lack of self-discipline was a consistent barrier across all classes of obesity. No differences were found across obesity classes I-III, with a pattern of similar self-reported ecological, psychosocial, and physical difficulties [33]. Regarding the difference in barriers by obesity class, the findings from this review are inconclusive, highlighting an understudied area needing further examination.

We found that the most frequently reported physical activity facilitators were the use of activity monitors (e.g., Fitbit) and social interaction/being part of a group. Participants stated that the monitors improved awareness of their activity level [26]. These participants also found the feedback from the monitors to be helpful [27], and the monitors were easy to use [26, 27]. Increases in physical activity among non-rural populations including older adults and adults with medical conditions (e.g., type 2 diabetes and musculoskeletal diseases) [34–37] and overweight and obesity, following participation in interventions using activity monitors for self-monitoring have been documented [38]. Such devices could enable individuals in rural communities with limited resources and amenities (e.g., recreational facilities), to independently track and be accountable for their physical activity.

While weight loss was not a primary facilitator in our review, it is notable that rural-dwelling African American women reported it as a motivator in one study [25]. Previous studies emphasize the importance of considering cultural norms [25] and unmet weight loss expectations [39] when aiming to increase motivation for exercise/physical activity. Therefore, prioritizing weight loss as the primary target of physical activity interventions is not recommended [40]. Instead, it is recommended to consider culturally-tailored physical activity interventions.

Our findings related to social interaction align with findings from a recent systematic review focused on adults with obesity [18] and a study among rural residing adults with overweight and obesity [19]. The findings from these articles highlight social support [18, 19], in addition to weight management [18], energy/physical fitness [18], and accessible and affordable fitness facilities as primary facilitators for physical activity [19]. We hypothesize that the social interaction motivation aspect is related to the lack of social support, a top barrier identified in our review. Social support is a multifaceted construct that has been found to be a key facilitator and determinant for physical activity adherence across adult populations underscoring its importance in intervention strategies [41–43].

Consistent with the Baillot et al. systematic review among adults with obesity [18], there were limited findings for physical activity preferences. Only one study included in this review reported intervention delivery preferences that were associated with individual characteristics (e.g., rurality, obesity) [28]. Results from the Baillot et al. review also showed variance in physical activity type, context and delivery preferences, across their included studies. Results indicated walking, water aerobics, cycling, swimming and rowing, dance or Zumba, and martial arts as the preferred physical activity types, with resistance training being less preferred by adults with obesity [18]. For context, there was preference for low- or no-cost physical activity interventions not solely focused on activity/exercise, structured routine. However, there was variation in social preferences with some participants preferring activities that could be done alone, and others favoring activities with people of similar ages and sex [18]. Given the overlapped focus on adults with obesity, our findings regarding preferences align closely with those of the review conducted by Short et al., which was included in both reviews [28]. Similar to social interaction/being part of a group facilitating physical activity reported earlier, we believe this preference for group interaction may be influenced by motivation from others [44]. Future research should explore the potential influence of the social interaction that occurs during sports and dance classes at local facilities on preferences for exercise location.

### Implications and Directions for Future Research

This review provides insights on the commonly reported physical activity barriers and facilitators cited by rural adults with obesity, an underserved, high-risk population in need of effective physical activity interventions. These findings have implications for healthcare professionals, researchers, and policy makers and local leaders. It is important that healthcare providers are cognizant of the barriers that individuals frequently encounter when recommending that their patients increase their physical activity. As for researchers, understanding the barriers and facilitators is key when designing future interventions for rural adult populations with obesity. Our findings regarding environmental barriers including the lack of sidewalks and bike lanes are applicable to policy makers and local leaders in rural communities. The existing policies that require or suggest walkways or bikeways in new public infrastructure projects to accommodate pedestrians [45], is inconsistently implemented in rural regions which warrants attention [46–48].

Although the American Heart Association (AHA)/American College of Cardiology (ACC)/ Obesity Society (TOS) has existing guidelines recommending that primary care practitioners evaluate and manage overweight and obesity in their patients [49], several challenges hinder effective treatment in rural areas. Limited healthcare access [50], health insurance challenges [51], low reimbursement rates [52], lack of specialized training in obesity medicine [53] makes it difficult to address obesity. Additionally, providers often fail to provide a diagnosis of obesity [54, 55] and even when a diagnosis is made, the limitations in specialized services [56] such as nutritionists, weight management professionals and obesity prevention programs can hinder necessary counselling and support. These limitations can impact the barriers, facilitators and preferences for physical activity among rural adult populations, particularly those with obesity. For instance, limited access to resources such as weight management professionals can lead to a lack of awareness about the importance of physical activity in relation to obesity creating a barrier to physical activity participation.

In response to these challenges in rural areas, the National Rural Health Association Rural Obesity and Chronic Disease Initiative Task Force has emphasized the need for state and national policies to include rural-specific programming ensuring that rural residents have access to obesity prevention and treatment services [54]. Recommended actions to support these initiatives consist of reintroducing the Halt Obesity in America Act, allowing rural residents to receive preventative obesity care, and the Improving Social Determinants of Health Act which mandates the Centers for Disease Control and Prevention (CDC) to establish a program aimed at improving health outcomes and reduce health inequities [54].

We were not able to draw conclusions related to physical activity preferences as only one included study reported on this aspect. Future research directions consist of assessing preferences (e.g., program type, duration, and time of day) [15] using mixed methods approaches as a qualitative approach would shed light on these preferences in addition to why certain aspects such as face-to-face interventions are preferred by some, but not others. This understanding would guide the development and design of future physical activity interventions.

### Rural U.S. Versus Other Countries

Despite the existing definition and classification of ‘rural’ in the U.S [57]., there is no wide-spread agreement on what constitutes rural [58]. For instance, in Australia, ‘rural and remote’ comprises all areas beyond major cities [59] whereas in Nepal, rural areas are ‘small-tight knit communities” lacking amenities and resources [60]. Even with an established definition and classification of rural in the U.S., rural areas within the US are heterogenous in many aspects (e.g., demographics, built environment, resources) [46–48] and the rural regions within other countries differ as well. Though most of the studies were conducted in the US, four were completed among adults residing in rural regions within Australia, Nepal, and Mexico. Given the heterogeneity across the countries in terms of demographics, built environment, resources, this introduces complexity for future research and physical activity programming, particularly in countries outside of the US. No themes or conclusions could be generated on barriers, facilitators, or preferences as each of the included studies from other countries provided findings for one category. Specifically, the study conducted in Mexico examined barriers [31], while the two studies from Australia focused on preferences [28], and barriers [29], and the study undertaken in Nepal delved into facilitators [30].

### Strengths and Limitations

This review is the first to identify barriers, facilitators, and preferences for physical activity among rural adult populations with obesity. Also, this review integrates qualitative and quantitative literature, allowing insights from interviews to complement numerical data. Interestingly, the representation of men was higher in comparison to previous obesity-focused reviews [18, 61]. However, there are limitations to note. Multiple articles were omitted as they were published in a language other than English. Participant characteristics were inconsistently reported; for example, information on prevalence of obesity, comorbidities, and average BMI, were not provided in some articles. This lack of detailed reporting hinders accurate representation of participants and applicability to the broader population. Most studies included in our review did not provide sufficient detail to differentiate barriers and facilitators specific to obesity versus coexisting comorbidities (e.g., diabetes, ischemic heart disease). While this limitation does not dimmish the value of our results – since obesity rarely occurs in isolation – it highlights the need for additional research to better tailor programs for individuals with both obesity and coexisting medical conditions.

## Conclusions

Effective physical activity interventions are essential due to the higher prevalence of physical inactivity, obesity, and preventable chronic health conditions in rural-dwelling adult populations. When developing these future interventions, it is important to consider and address barriers and facilitators, especially factors such as built environment, use of activity monitors and social interaction. Future research should aim to explore physical activity preferences of rural adults with obesity and examine how barriers, facilitators, and preferences can vary across obesity classes to further advance this field.

## Figures and Tables

**Table 1:** Characteristics of included studies (*N* = 10)

Author, year, country [ref]	Design & Methods	Participant Characteristics (Sample size (*n*), Age, Gender, BMI (mean, (SD)), Obesity (%), Comorbidities	Rural Characterization, BMI Comparisons, Rural Comparison (if not purely rural)	Intervention Characteristics	Barriers	Facilitators	Preferences
Adachi-Mejia, AM., 2017, United States [22]	Telephone Interview	**N** = 2,025; **Age**: 57.82 (15.57); **Gender**: female (60%); **BMI** = 26.97 (5.26) **percent obese** = not provided; **Comorbidities**: not assessed	**Rural characterization**: “nine small towns located within micropolitan statistical areas in Washington, Texas, and the Northeast (New Hampshire and New York); **BMI comparisons?** Yes – barriers	Not applicable	Difficulty walking in relation to environment was associated with higher BMI in all regions (Northeast, Texas, and Washington); Environmental barriers/concerns (unattended dogs) were associated with higher BMI in Northeast	Not assessed	Not assessed
Short, CE. 2014, Australia [28]	Telephone Interview	**N** = 1,261; **Age**: 52.79 (16.31); **Gender**: male (50%), female (50%); **BMI** = 30.03 (14.67); **percent obese** = 30%; **Comorbidities**: unspecified chronic illness (46%)	**Rural characterization**: 22% rural, 52% city, 26% town; **BMI comparisons?** Yes- preference (delivery mode); **Rural comparisons?** Yes, city and town	Not applicable	Not assessed	Not assessed	**Face-to-face** preference with an instructor was strongly associated with obesity. **Web-based intervention** preference positively associated with living in rural area but negatively associated with obesity. **Print-based intervention** preference was negatively associated with obesity. **Group-based program (with instructor)** preference negatively associated with obesity
Jilcott Pitts, SB.,2015, United States [24]	Self- and interviewer-administered measures; Anthropometrics	**N** = 366; **Age**: 55; **Gender**: 76% female; **BMI** = 35.9 (9.4); **percent obese** = Not mentioned; **Comorbidities**: not assessed	**Rural characterization**: Rural eastern North Carolina; **BMI comparisons?** Yes- associations with neighborhood barriers and PA behaviors	Not applicable	More perceived neighborhood-level barriers associated with higher BMI	Not assessed	Not assessed
Parajuli, J., 2014, Nepal [30]	Interviewer administered questionnaire; Anthropometrics	**N** = 385, **Age**: 54.4 (11.5); **Gender**: 51.4% female; **BMI** = 24.26 (3.33); **percent obese** = 48%; **Comorbidities**: Type II diabetes	**Rural characterization**: rural, urban, semi-urban (distributions not mentioned); **BMI comparisons?** No; **Rural comparisons?** Yes, urban, semi urban	Not applicable	Not assessed	Participants with positive family history of diabetes and higher socioeconomic class were more adherent to PA	Not assessed
Warren, JC., 2017, United States [25]	Self-administered measures; Questionnaires	**N** = 497; **Age**: 52.68 (12.37); **Gender**: Female (72.2%); **BMI**: not specified; **precent obese** = 65% **Comorbidities**: hypertension only (46.5%); diabetes only (7.4%); diabetes and hypertension (46.1%),	**Rural Characterization**: “rural South”; **BMI comparisons?** No	Not applicable	Not assessed	Those who were motivated for weight loss but not for increasing exercise twice as likely to be obese, African Americans were 1.7 times as likely to be motivated for weight loss, not exercise.	Not assessed
Harrison, C., 2017, Australia [29]	Self-administered questionnaires; Anthropometrics	**N** = 649; **Age**: 39.6(6.7); **Gender**: female (100%); **BMI** = 28.8(6.9); **percent obese** = 34% **Comorbidities**: hypertension, PCOS, previous gestational diabetes.	**Rural characterization**: Rural community in Australia; **BMI comparison?** Yes, compared across BMI categories (healthy, overweight, and obese)	Not applicable	Higher BMI was associated with reduced social support from friends, negative PA environment perceptions (lack of pleasant places to be active)	Not assessed	Not assessed
Zavala, G., 2022, Mexico [31]	Self-reported data from publicly available data	**N** = 6,406; **Age**: median 39, IQR (30–47); **Gender**: female (68.1%); **BMI**: normal (25.0%), overweight (38.4%), obesity (36.6%)	**Rural characterization**: 50.5% “less than 2500 inhabitants in the town” **Rural comparison?** Yes, urban (49.5%) “more than 2500 inhabitants in the city or town	Not applicable	Lack of motivation was the barrier with the largest difference between overweight/obese and the healthy BMI group	Not assessed	Not assessed
Adachi-Mejia, AM., 2016, United States [23]	Self-administered survey (close ended and open-ended questions); Anthropometrics	**N** = 78; **Age**: 52.8 (14.5); **Gender**: Female (100%); **BMI** = 35.4 (9.2); **percent obese** = 75.6%; **Comorbidities**: Not assessed	**Rural characterization**: 100% rural (New Hampshire and Vermont); **BMI comparisons?** Yes, pre-obese, and classes I-III	Not applicable	**Close-ended survey approach**: *Participants with obesity reported* – Lack of self-discipline **Open-ended survey approach**: Most common across *all classes of obesity* – knee issues, depression, lacking company, weather (too hot), lacking time and work. *Class I:* ankle injury, asthma, weight, back issues, chronic illness, hip issues, low energy, pain depression, mood, prefers doing other things, care duties, family demands. *Class II:* asthma, weight, dislike exercise, migraines, procrastination, lazy *Class III:* asthma, weight, exhaustion, diabetes, doesn’t see results, out of shape, lazy, obligations	Not assessed	Not assessed
Eisenhauer, CM., 2017, United States [26]	Self-administered survey; Semi-structured interview	**N** = 12; **Age**: 50.9; **Gender**: 100% male; **BMI** = 34.1; **percent obese** = 75%; **Comorbidities**: Hypertension	**Rural characterization**: Rural Northern Plains state; **BMI comparisons?** No.;	3-week pilot study Participants used Fitbit activity tracker to log food intake and physical activity. Participants received text messages 1–3 times per day	Technological disparities; poor technology infrastructure; seasonal fluctuations in PA demands (i.e., occupational, religious, and recreational); resource access; fatigue	Being part of a group; activity monitor	Not assessed
Batsis, JA., 2016, United States [27]	Self-administered measures; Semi-structured interviews	**N** = 8; **Age**: 73.4 (4); **Gender**: female (50%); **BMI** = 34.5 (4.5); **percent obese** = 100% **Comorbidities**: hypertension (75%), dyslipidemia (63%), diabetes (38%), osteoarthritis (50%), and sleep apnea (50%)	**Rural characterization**: geriatric primary care practice at a rural academic medical center; **BMI comparisons?** No	3-week feasibility and acceptability trial Participants were provided a Fitbit activity tracker followed by a demonstration. Week 1: participants received a 10-minute phone call for encouragement and to troubleshoot use of Fitbit activity tracker. Week 4: participants returned activity tracker and completed study questionnaires	Lack of time/finding time	Feedback and self-monitoring; motivation from fitness tracker; “better” weather (i.e., late spring or summer), and environment; group engagement; social interaction	Not assessed

Abbreviations: BMI, body mass index; IQR, interquartile range; PA, physical activity; PCOS, Polycystic Ovary Syndrome

## Data Availability

No datasets were generated or analysed during the current study.
